# Predicting Surgical Outcome in Drug-Resistant Epilepsy by Combining Interictal Biomarkers within a Machine Learning Framework

**DOI:** 10.21203/rs.3.rs-8682213/v1

**Published:** 2026-02-17

**Authors:** Hmayag Partamian, Saeed Jahromi, M Scott Perry, Eleonora Tamilia, Joseph R. Madsen, Jeffrey Bolton, Scellig Stone, Phillip Pearl, Christos Papadelis

**Affiliations:** Cook Children’s Health Care System; Cook Children’s Health Care System; Cook Children’s Health Care System; Boston Children’s Hospital; Boston Children’s Hospital; Boston Children’s Hospital; Boston Children’s Hospital; Boston Children’s Hospital; Cook Children’s Health Care System

**Keywords:** interictal epilepsy biomarkers, epilepsy surgery, combined interictal features, supervised machine learning

## Abstract

Delineating the epileptogenic zone (EZ) is essential for achieving seizure freedom in drug-resistant epilepsy (DRE). Conventionally, seizure onset derived from ictal intracranial EEG (iEEG) approximates the EZ, but acquiring ictal data can be challenging. Interictal iEEG abnormalities offer abundant, easily acquired, non-seizure-dependent markers of the epileptogenic tissue; however, these biomarkers offer limited specificity. Here, we propose a machine-learning framework that integrates interictal spike and ripple features to automatically delineate the EZ and predict outcome with improved performance compared to individual biomarkers. We retrospectively analyzed iEEG data from 62 children with DRE ([34 good (Engel 1) outcomes] undergoing neurosurgery, automatically detected spikes and ripples, and computed temporal, spectral, and spatial features for each channel. We trained Random Forest classifiers to predict the EZ using combinations of these features. The predicted EZ derived from spike-based and combined spike-ripple feature sets outperformed those from individual biomarkers in defining the EZ, with an area under the receiver operating characteristic curve of 0.9 and 74% spatial overlap with resection. Although most individual features and classifiers predicted the outcome, the combined feature model performed best (i.e., sensitivity 88%, specificity 68%, and accuracy 79%). Our findings demonstrate that integrating multimodal interictal features improves the EZ delineation, providing valuable prognostic insights for epilepsy surgery.

## Introduction

Precise delineation of the epileptogenic zone (EZ) remains one of the most significant challenges for surgery planning and achieving seizure freedom post-surgery in patients with drug-resistant epilepsy (DRE).^1,2^ This hypothetical zone is often approximated by the seizure onset zone (SOZ), which is identified by manual review of ictal activity captured by intracranial EEG (iEEG) to detect the brain region(s) that initiate clinical seizures.^1^ Yet, increasing evidence suggests that interictal electrophysiological abnormalities observed in iEEG recordings (i.e., brief pathological events occurring between seizures) may also approximate the EZ.^3,4^ Importantly, identifying such biomarkers from readily available interictal data offers a significant advantage by eliminating the need to wait for a seizure to occur.^5,6^

Among the proposed interictal epilepsy biomarkers, spikes and high-frequency oscillations (HFOs) are the most extensively studied.^3,4,7^ Interictal spikes, brief high-amplitude deflections typically occurring in the 1–70 Hz frequency band, are routinely used in clinical practice as markers of the irritative zone.^6,8,9^ HFOs, which are considered more specific biomarkers of the EZ compared to spikes^10,11^, are commonly classified into ripples (80–250 Hz) and fast ripples (250–500 Hz).^12–14^ Fast ripples, although closely linked to the EZ^15–17^, are less frequently observed in patients with DRE, and their low sensitivity may limit their value for the EZ localization.^18,19^ On the other hand, ripples are present in most patients with DRE but are also observed in physiological brain regions.^6,10,20,21^ Thus, the presurgical value of interictal biomarkers, such as the spikes and HFOs, as biomarkers of the EZ remains largely debated since the cortical areas that generate them often extend beyond the EZ into brain regions that do not need to be resected or ablated for the patient to become seizure-free.^11,20,22,23^

Because of these limitations, temporal (i.e., timing and temporal occurrence), spectral (i.e., frequency content), and spatial (i.e., distribution across brain regions) features of spikes and ripples have been examined as potential indicators of epileptogenicity. In the temporal domain, the spike and ripple rates have been linked to the underlying brain tissue’s epileptogenicity.^22,24,25^ Yet, spike-generating regions correspond to the broader irritative zone, which can extend beyond the EZ and overlap with physiological brain regions.^26,27^ Moreover, not all ripples are pathological, as physiological ripples also occur, particularly in brain regions such as the hippocampus, occipital cortex, and paracentral areas.^28–30^ Consequently, spike and ripple rates alone are unreliable predictors of the EZ. Recent studies have shown that the temporal co-occurrence of spikes and ripples may predict the EZ more effectively, or with comparable efficacy, compared to spikes or ripples alone.^22,25,31^ This improved performance likely reflects the co-occurrence of spikes and ripples as a more pathologic pattern of neuronal synchronization than either event in isolation.^32^ Yet, the interpretation of ripples remains challenging due to their susceptibility to contamination by artifacts arising from the Gibbs phenomenon, particularly in recordings containing epileptiform spikes.^33,34^ In the spectral domain, frequency-based features have been shown to identify abnormal oscillatory activity associated with epileptogenic networks.^35–37^ However, they are limited by their sensitivity to sensor noise and biological artifacts.^38,39^ Spatial analyses have demonstrated that spikes and ripples often originate in specific brain regions and propagate to adjacent and distant areas within a few tens of milliseconds.^10,11,40,41^ Recent studies have also shown that the onset generator of spikes and ripples (i.e., regions where discharges show the earliest peaks) are correlated with the EZ,^10,40,42,43^ often outperforming the clinically defined SOZ in predicting the surgical outcome.^11,40,44^ However, the spatial distribution of spikes and ripples fluctuates over time, shifting among multiple regions rather than arising from a single consistent source.^45,46^ Therefore, no single biomarker fully defines the EZ; rather, each reflects only a partial and variable aspect of epileptogenicity, highlighting the need for complementary approaches.

It is reasonable to presume that the integration of temporal, spectral, and spatial features of interictal biomarkers will yield a more comprehensive and precise delineation of the EZ.^47–49^ Yet, the practical implementation of this approach is technically challenging. iEEG recordings are often performed with several dozens of contacts sampled at high rates (> 1 kHz) and collected over several hours. As a result, they generate a massive amount of data, making the process time-consuming, labor-intensive, and prone to bias and error. This underscores the need to develop novel automated approaches that integrate the complementary strengths of these biomarkers and features into a unified framework, enabling a more objective, efficient, and accurate characterization of the EZ.

Machine learning (ML) offers a robust framework for automating the analysis of large-scale data and integrating heterogeneous, high-dimensional features that are difficult to analyze manually or with conventional statistical methods.^50,51^ Previous studies have incorporated temporal, spectral, and spatial features using ML to delineate the EZ in patients with DRE.^37,52–59^ However, few studies have further compared the performance of individual features with multi-feature ML-based frameworks’ findings.^53,60^ This comparison is critical, as the epileptogenic brain regions identified by individual spike and ripple features may differ across patients, or map incompletely to the EZ or extend beyond it. These limitations highlight the need for methods that systematically integrate heterogeneous biomarkers for EZ prediction. ML-based frameworks are well-suited for this task, as they can fuse multiple feature types, thereby improving accuracy, robustness, and interpretability in EZ mapping and surgical outcome prediction.

In this study, we propose an ML-based multimodal framework that integrates diverse interictal spike and ripple temporal, spectral, and spatial features to automatically identify the EZ and predict postsurgical outcome in patients with DRE. We hypothesize that the proposed framework outperforms single-feature analyses in delineating the EZ and predicting the outcome by capturing nonlinear and cross-domain relationships among epileptogenic biomarkers. To evaluate this framework, automatic spike and ripple detectors were applied to iEEG recordings from children and young adults with DRE to extract multidomain electrode-level features. These features were then used to train Random Forest classifiers, with different combinations of features as predictors and resection as the target, to define the predicted EZ (PEZ). The PEZs derived from individual spike and ripple features and from the proposed feature-combining approach were analyzed to evaluate their ability to predict the EZ. We then assessed whether resection of each PEZ could predict surgical outcome. By combining multimodal interictal features, this study establishes a data-driven framework for more accurate localization of the EZ and prediction of surgical outcome for patients with DRE.

## Results

### Patient cohort

We retrospectively analyzed interictal iEEG data from 62 children and young adults (29 females) with focal onset DRE, having either a good (34 patients, Engel I) or poor (28 patients, Engel >I) surgical outcome following epilepsy surgery [median follow-up: 2.5 years (2–5)], who satisfied the study’s inclusion and exclusion criteria (see Materials and Methods). Patient demographics are summarized in Table 1. The patients had a median age at surgery of 12 years (range: 8–15.5) with a median seizure onset age of 4.5 years (2–9). Our dataset included patients with three implantation types: (i) subdural electrodes [electrocorticography (ECoG)] (13 patients; eight with good outcome); (ii) stereotactic EEG (sEEG) implantation (36 patients; 20 with good outcome); and (iii) both subdural and depth electrodes (13 patients; 6 with good outcome). The median number of implanted contacts was 124 (102–151). Seventeen patients (27.4%) had non-lesional MRI, 34 patients (54.8%) had a malformation of cortical development, and 11 patients (17.8%) had acquired brain injury. Twenty-six patients (41.9%) had temporal lobe epilepsy. No significant differences in these characteristics were found between good and poor outcome patients (Table 1).

### Overall processing Pipeline

**Figure 1:**
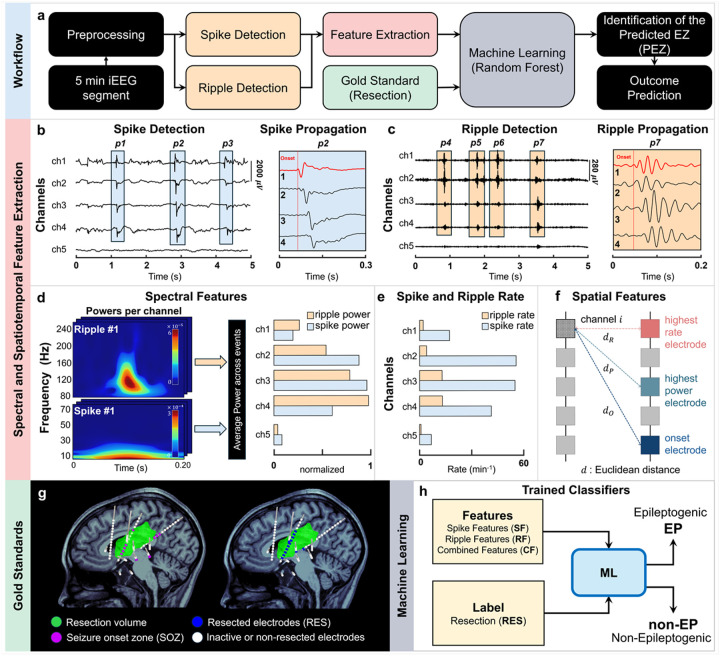
Processing pipeline of the automatic epileptogenic zone (EZ) classifier. **a** Five-minute interictal iEEG segments were preprocessed, cleaned, and filtered in two frequency bands: spike band (1–70 Hz) and ripple band (80–250 Hz). Automatic spike and ripple detectors identified spike and ripple events, from which temporal, spatial, and spectral features were extracted. The seizure onset zone (SOZ) and resection zone (RES) were defined based on clinical reports and postoperative MRI-defined resection volumes. Multiple Random Forest classifiers were trained using different feature and label sets for mapping the predicted EZ (PEZ), and the correspondence between the PEZ and surgical outcome was evaluated. **b** In the spike band, the automatic spike detector identified spikes (blue). Detected propagation events (*p1–p3*) were analyzed to compute a spike propagation index per channel. Channels exhibiting earliest activation within each event were defined as onset spikes (red signal in *p2*). **c** In the ripple band, the automatic ripple detector identified ripples (yellow). Similar propagation analysis of detected events (*p4–p7*) yielded a ripple propagation index per channel, and channels showing earliest activation were defined as onset ripples (red signal in p7). **d** Spectral power features were computed for spikes and ripples across all channels and normalized within [0,1]. **e** Spike and ripple rates were calculated per channel to characterize interictal activity. **f** Spatial distribution features were derived using Euclidean distances between each channel involved in the events and the contacts with highest event rate, maximum power, and onset activity. **g** Coregistrationof pre- and postoperative MRI with CT scans containing intracranial implantations was used to localize iEEG electrode coordinates and delineate the resected volume from postoperative MRI, which served as the gold standard for epileptogenic zone (EZ) prediction, with the contacts located within the resection defined as resected contacts (RES). **h** Multiple Random Forest classifiers were trained using three different feature sets [spike features (SF), ripple features (RF), and combined features (CF)] to classify contacts as epileptogenic (EP) or non-epileptogenic (non-EP).

The proposed framework automatically identifies the EZ from interictal iEEG data through automated detection of spikes and ripples, spike and ripple features extraction, and ML (Fig. 1). We initially preprocessed a 5-minute interictal iEEG segment to remove artifacts and noisy channels (Fig. 1a). The data were then bandpass filtered into the spike (1–70 Hz)^40^ and ripple (80–250 Hz)^44^ frequency bands. Spikes and ripples were detected using previously validated fully automated algorithms.^10,62^ From the detected spikes and ripples, we extracted a set of temporal, spectral, and spatial features. For the delineation of the EZ, we used as gold standard the resected volume of patients with good surgical outcome: channels inside the resection were labeled as EZ, and those outside as non-EZ. We first evaluated the individual discriminatory power of each feature for classifying the EZ vs. non-EZ channels. We then trained Random Forest classifiers to automatically identify the predicted EZ (PEZ). Finally, we assessed whether resecting the PEZ, which was mapped by each feature and the classifiers, could predict surgical outcome (Fig. 1a).

To characterize the epileptogenic properties of each channel, we derived a set of temporal, spectral and spatial features from the detected spikes and ripples (Fig. 1b–f). Using in-house algorithms, we first identified propagation sequences of both spike and ripple events (Fig. 1b–c). For each type of event, we determined the onset and spread channels and derived multiple quantitative features per channel, including propagation indices (Fig. 1b–c), power (Fig. 1d), spatial distribution (Fig. 1e), and spike and ripple rates (Fig. 1f). Finally, we computed the co-occurrence of spikes and ripples for each channel (see Materials and Methods). Table 2 summarizes the features adopted in this study. The selected features were designed to capture complementary temporal, spectral, and spatial characteristics of interictal spikes and ripples, known to reflect epileptogenic tissue. Spike and ripple rates (i.e. SR and RR, respectively) and power (i.e. SP and RP, respectively) quantify local excitability and pathological high-frequency activity^63,64^, while spike and ripple propagation indices (i.e. SPI and RPI, respectively) characterize onset and spread channels.^65^ The spatial distribution of interictal epileptiform events has been shown to reflect underlying epileptogenic organization and network spread, with events clustering around putative onset regions and dispersing along pathological propagation pathways.^48^ We used several distance-based measures to quantify the spatial distribution of epileptiform events relative to the contact with the highest spike and ripple rate (i.e. ED-HSR and ED-HRR, respectively), the highest spike and ripple power (i.e. ED-HSP and ED-HRP, respectively), and the spike and ripple onset (i.e. ED-SO and ED-RO, respectively) contacts. In addition, we included the spike–ripple co-occurrence (SRC) to quantify pathological coupling between spikes and ripples, which reflects abnormal local network synchronization and has been strongly associated with epileptogenicity.^22,65^ Together, these features integrate local and network-level information, providing a physiologically grounded and interpretable representation for automated delineation of the EZ.

We next coregistered the pre- and post-operative MRIs with the electrode implantation computerized tomography (CT) to map the locations of iEEG electrodes and the resected brain regions (Fig. 1g). Pediatric epileptologists defined the SOZ through visual inspection of ictal iEEG data independently from this study. We finally trained Random Forest classifiers using different subsets of the extracted features as predictors: spike features alone (SF), ripple features alone (RF), and combined spike–ripple features (CF). Resected contacts were labeled as epileptogenic, and all remaining contacts were considered non-epileptogenic (Fig. 1h). The contacts predicted as epileptogenic defined the PEZ. Training was performed only on good outcome patients, as their resections were assumed to contain the actual EZ, and model performance was evaluated using leave-one-patient-out cross-validation (LOPO-CV). We then quantified the PEZ with spatial measures: (i) focality (*F*_*PEZ*_); (ii) overlap with resection (*O*_*RES*_), and (iii) average distance from resection (*D*_*RES*_).^37^ We defined the *F*_*PEZ*_ as the average Euclidean distance (in mm) between the PEZ contacts. We defined the *O*_*RES*_ as the percentage of PEZ contacts within 10 mm of resection. We defined the *D*_*RES*_ as the average Euclidean distance of the PEZ contacts to the resection. While training was performed using only good outcome patients, testing included both good and poor outcome patients. Finally, we tested whether the resection of the PEZ was associated with surgical outcome using the *O*_*RES*_ as predictor. Additional analyses compared the performance of individual features and classifiers trained on different feature subsets in predicting the EZ and outcome. We also assessed how feature selection and implantation type impacted the accuracy of EZ and outcome prediction (see Materials and Methods).

### Predictive power of individual features

**Figure 2:**
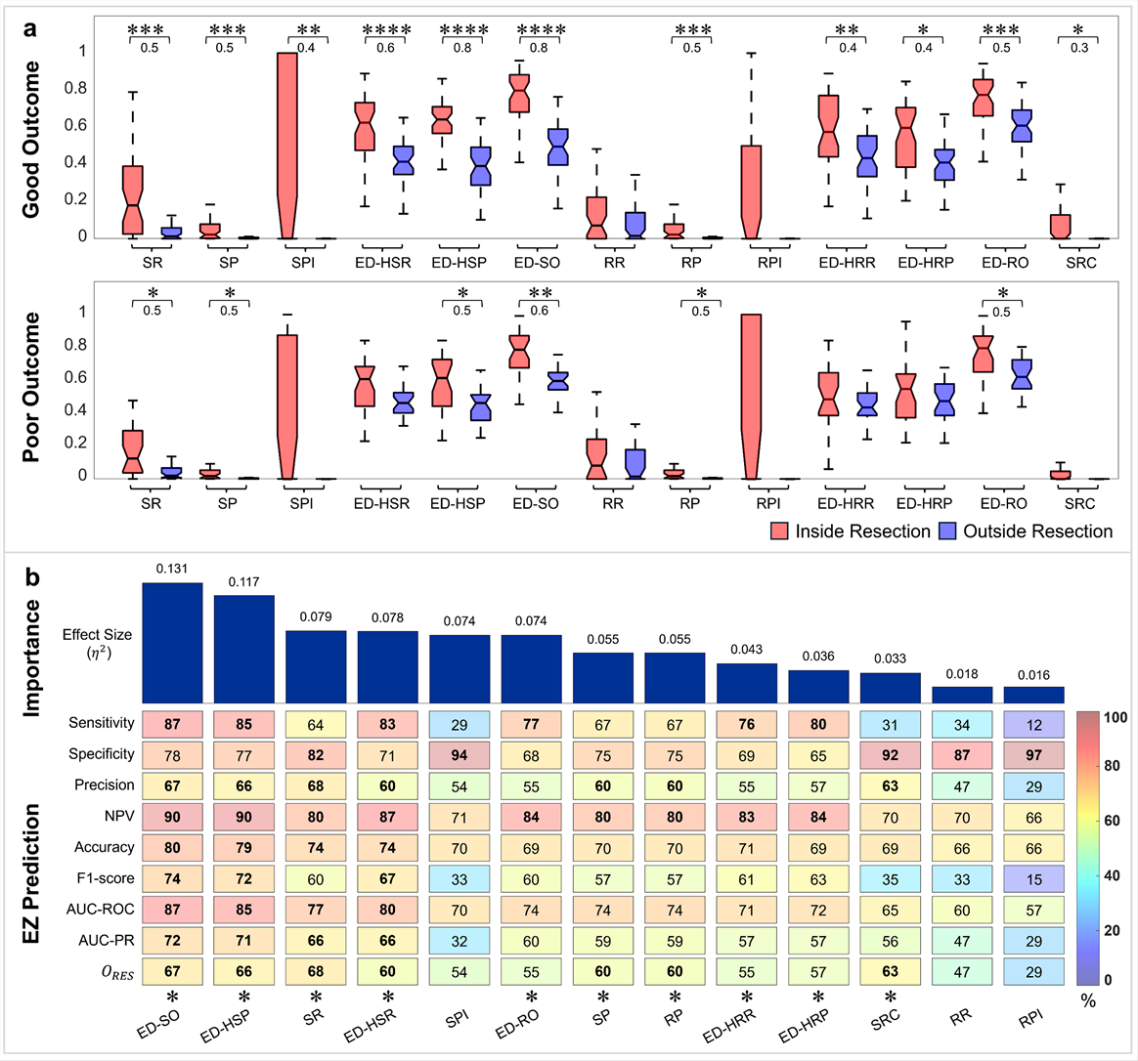
Predictive power of individual features. **a** Comparison of feature values inside versus outside the resection zone in good and poor outcome patients. For each feature, the red box plot represents the median feature value inside the resection, and the blue box plot represents the median value outside the resection. The notches indicate the median across all patients, while the lower and upper box edges correspond to the 25^th^ and 75^th^ percentiles. Whiskers extend to the minimum and maximum values after omitting outliers. Significant differences are indicated by asterisks: *P < 0.05, **P < 0.01, ***P < 0.001, ****P < 0.0001 (*Wilcoxon signed-rank* test, significance levels were corrected for multiple comparisons using the Bonferroni method). The number displayed below the asterisks represents the effect size. **b** Ranked importance (effect size) and performance of individual features in predicting the resection (RES) in good outcome patients. Performance measures were evaluated using Youden index–based optimal thresholding with leave-one-patient-out cross-validation (LOPO-CV). Asterisks denote statistical significance of features in predicting surgical outcome based on the *Fisher* test (*P < 0.05). Features: SR (spike rate), SP (spike power), SPI (spike propagation index), ED-HSP (distance from highest spike power), ED-HSR (distance from highest spike rate), ED-SO (distance from spike onset), RR (ripple rate), RP (ripple power), RPI (ripple propagation index), ED-HRP (distance from highest ripple power), ED-HRR (distance from highest ripple rate), ED-RO (distance from ripple onset), SRC (spike-ripple co-occurrence).

To assess the relationship between the extracted features and the EZ, we compared the normalized feature values inside vs. outside resection in good and poor outcome patients, separately. In good outcome patients, we observed increased SR (*P* < 0.001), SP (*P* < 0.001), SPI (*P* < 0.01), ED-HSR (*P* < 0.0001), ED-HSP (*P* < 0.0001), ED-SO (*P* < 0.0001), RP (*P* < 0.001), ED-HRR (*P* < 0.01), ED-HRP (*P* < 0.05), ED-RO (*P* < 0.001), and SRC (*P* < 0.05) inside (compared to outside) resection (Fig. 2a). In poor outcome patients, we observed higher SR (*P* < 0.05), SP (*P* < 0.05), ED-HSP (*P* < 0.05), ED-SO (*P* < 0.01), RP (*P* < 0.05), and ED-RO (*P* < 0.05) inside (compared to outside) resection (Fig. 2a). To assess the predictive power of each feature, we computed their performance in predicting the EZ in good outcome patients (Fig. 2b). First, we evaluated feature importance using one-way ANOVA by ranking the proportion of variance (η^2^) explained by class labels (epileptogenic vs. non-epileptogenic) in decreasing order (see Materials and Methods). The top-ranked features ED-SO, ED-HSP, SR, ED-HSR, SPI, and ED-RO exhibited strong discriminative power in differentiating between epileptogenic and non-epileptogenic contacts (η^2^>0.06) (Fig. 2b). Next, we trained single-feature threshold-based classifiers and their performance in discriminating between epileptogenic and non-epileptogenic contacts using different performance metrics including sensitivity, specificity, precision, negative predictive value (NPV), accuracy, F1-measure, as well as the area under the receiver operating characteristic curve (AUC-ROC), precision–recall curve (AUC-PR), and *O*_*RES*_ (see Materials and Methods). Among all features, SR, ED-HSR, ED-HSP, and ED-SO achieved the highest AUC-ROC and AUC-PR (> 74% and 66%, respectively) and exhibited > 60% overlap with resection. The resection of the PEZ, estimated from most single-feature classifiers, also predicted outcome, except for the SPI, RR, and RPI (Fig. 2b).

### Predictive power of feature sets

**Figure 3:**
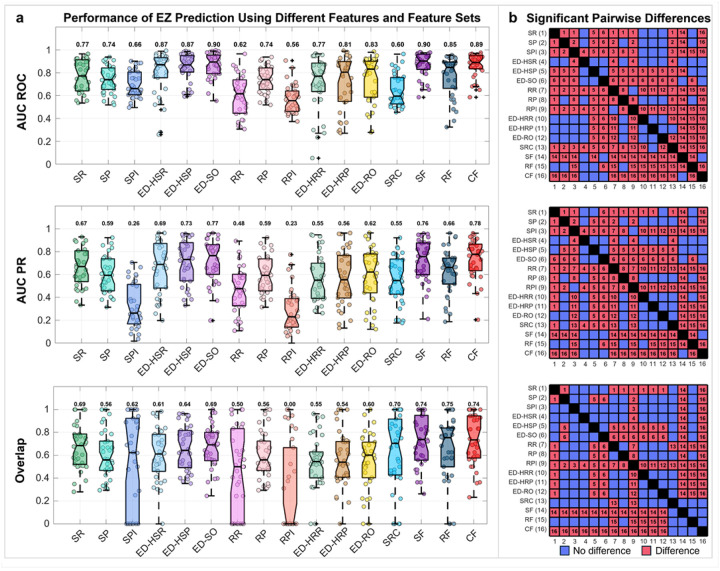
Predicting the epileptogenic zone (EZ) using individual features and different feature sets. **a** Performance metrics were evaluated for different classifiers in predicting the EZ (PEZ) using individual features and spike features (SF), ripple features (RF), and combined features (CF). Metrics included area under the receiver operating characteristic curve (AUC-ROC), area under the precision–recall curve (AUC-PR), and the overlap with resection (). Classification performance was assessed using a leave-one-patient-out cross-validation (LOPO-CV) approach trained on good outcome patients. The notches represent the median metric value across all patients, while the lower and upper box edges correspond to the 25^th^ and 75^th^ percentiles. Whiskers extend to the minimum and maximum values after excluding outliers. The number above each boxplot represents the median value. **b** Pairwise comparison of AUC-ROC, AUC-PR, and for epileptogenic zone prediction, derived from individual interictal features and machine-learning classifiers using SF, RF, and CF as predictors. Each red cell indicates a significant difference (*P* < 0.05, *Wilcoxon signed-rank* test, FDR corrected) between the corresponding pair of models, with the ID of the better-performing model (higher median AUC-ROC, AUC-PR, and, respectively) displayed inside the cell. Blue cells indicate no differences. Features: SR (spike rate), SP (spike power), SPI (spike propagation index), ED-HSP (distance from highest spike power), ED-HSR (distance from highest spike rate), ED-SO (distance from spike onset), RR (ripple rate), RP (ripple power), RPI (ripple propagation index), ED-HRP (distance from highest ripple power), ED-HRR (distance from highest ripple rate), ED-RO (distance from ripple onset), SRC (spike-ripple co-occurrence).

The performance of the Random Forest classifiers using different feature sets (i.e., SF, RF, CF) as predictors of the EZ was evaluated using the LOPO-CV, AUC-ROC, AUC-PR, and *O*_*RES*_ computed for individual features and feature sets (see Materials and Methods). We found that SF and CF achieved higher AUC-ROC (0.9 and 0.89, respectively) compared to most individual features, except for the ED-SO (0.9). In terms of AUC-PR, SF, CF, and ED-SO showed higher values compared to all individual features (AUC-PR = 0.76, 0.78, and 0.77, respectively). Many individual features, however, showed higher AUC-ROC and AUC-PR compared to RF. Regarding overlap with resection, SF, RF, and CF all achieved *O*_*RES*_ > 74%, which was higher than the individual features (*O*_*RES*_ ≤ 70%) (Fig. 3a).

Statistical pairwise comparisons between the performance measures of individual features and classifiers showed that SF and CF outperformed all individual features in terms of AUC-ROC and AUC-PR, except for the ED-HSR, ED-HSP, and ED-SO. RF showed higher performance than some individual features but did not exceed SF or CF. No differences were observed between SF and CF for the AUC-ROC and AUC-PR. Regarding the *O*_*RES*_, SF and CF outperformed all individual features except the SRC, while no differences were observed among the SF, RF, and CF (Fig. 3b).

### Effect of implantation type on EZ predictability

To determine whether the implantation type (i.e., ECoG, sEEG, or both subdural and depth electrodes) influenced the PEZ’s predictive power, we trained Random Forest classifiers separately for cohorts sharing the same implantation type. For good outcome patients, we estimated the PEZ using SF, RF, and CF and then calculated the AUC-ROC, AUC-PR, and *O*_*RES*_ (see Materials and Methods). We found no differences between the AUC-ROC, AUC-PR, and *O*_*RES*_ of the PEZs with resection among the different implantation types for any of the feature sets (see **Supplementary Note 2**).

### Focality, proximity, and overlap of PEZ with resection

**Figure 4:**
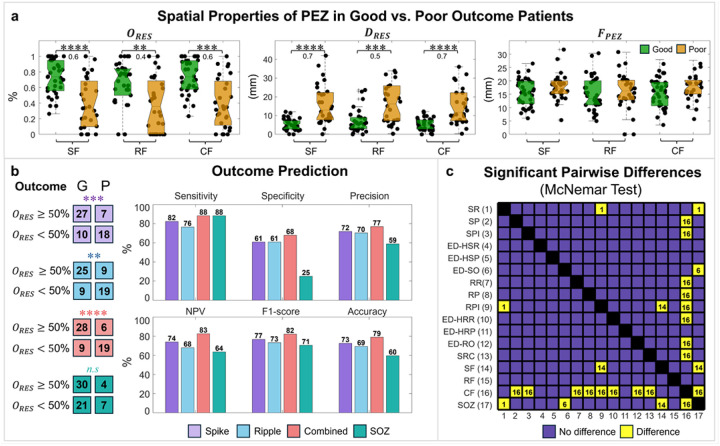
Spatial properties of the predicted epileptogenic zone (PEZ) and outcome prediction. **a** Overlap with resection (, %), distance from resection (, mm), and focality (, mm) were computed for PEZ identified by classifiers trained using spike features (SF), ripple features (RF), and combined features (CF) as predictors for good- and poor outcome patients. The notches indicate the median values across all patients, while the lower and upper box edges correspond to the 25^th^ and 75^th^ percentiles. Whiskers extend to the minimum and maximum values after omitting outliers. Significant differences are indicated by asterisks: *P < 0.05, **P < 0.01, ***P < 0.001, ****P < 0.0001 (*Wilcoxon rank-sum* test, significance levels were corrected for multiple comparisons using the Bonferroni method). **b** Outcome prediction based on resection of the PEZ. Confusion matrices show outcome prediction when 50% of the PEZ was resected. The PEZ was predicted using spike features (SF), ripple features (RF), and combined features (CF). The seizure onset zone (SOZ) was also assessed for outcome prediction. Evaluation metrics included sensitivity, specificity, precision, negative predictive value (NPV), F1-score, and accuracy. Significant differences are indicated by asterisk above the confusion matrices: *P < 0.05, **P < 0.01, ***P < 0.001, ****P < 0.0001 (*Fisher’s exact* test). **c** Pairwise comparison of individual feature and feature subset-based classifiers (SF, RF, and CF) outcome prediction using the McNemar test. Each yellow cell indicates a significant difference (p < 0.05, *McNemar* test) between the corresponding pair of models, with the ID of the better-performing model (higher average prediction) displayed inside the cell. Blue cells indicate no differences. Feature sets: SF (spike features), RF (ripple features), CF (combined features). Features: SR (spike rate), SP (spike power), SPI (spike propagation index), ED-HSP (distance from highest spike power), ED-HSR (distance from highest spike rate), ED-SO (distance from spike onset), RR (ripple rate), RP (ripple power), RPI (ripple propagation index), ED-HRP (distance from highest ripple power), ED-HRR (distance from highest ripple rate), ED-RO (distance from ripple onset), and SRC (spike–ripple co-occurrence).

To assess the concordance of PEZ with RES, we compared *O*_*RES*_, *D*_*RES*_, and *F*_*PEZ*_ across patients with good and poor outcomes for the three feature subsets. We found that the SF, RF, and CF had higher *O*_*RES*_ and lower *D*_*RES*_ in good compared to poor outcome patients (*P* < 0.01, *d ≥ 0.4*). No differences in *F*_*PEZ*_ were observed between good and poor outcome patients for any of the feature sets. Notably in good outcome patients, the SF had a median *O*_*RES*_ *≈* 74 (56–95)%, RF had a median *O*_*RES*_ *≈* 75 (50–85)%, and CF had a median *O*_*RES*_ *≈* 74 (57–94)% (Fig. 4a).

### EZ and Surgical Outcome Prediction Across Features and Classifiers

To evaluate the PEZ as an interictal biomarker of the EZ, we examined its ability to predict surgical outcome (see Materials and Methods). We found that all PEZ derived from SF, RF, and CF predicted outcome (*P* < 0.01, *Fisher Exact* test), while the clinically defined SOZ did not predict outcome (Fig. 4c). Additionally, the CF model predicted outcome with the highest sensitivity (88%), specificity (68%), precision (77%), NPV (83%), F1-score (82%), and accuracy (79%) (Fig. 4c). We finally performed a pairwise McNemar test on the confusion matrices of outcome predictions of individual features, feature sets (i.e., SF, RF, CF), and the SOZ, to determine whether their outcome predictions were significantly different (Fig. 4c). We found that SR was better than RPI and the SOZ, and ED-SO was better than the SOZ (*P* < 0.05, *McNemar* test). SF showed better predictive power compared to the RPI and the SOZ (*P* < 0.05, *McNemar* test). Finally, CF had higher predictive power of surgical outcome than all individual features except the SR, ED-HSR, ED-HSP, ED-SO, ED-HRP, SF, and RF (*P* < 0.05, *McNemar* test).

### Representative cases

**Figure 5:**
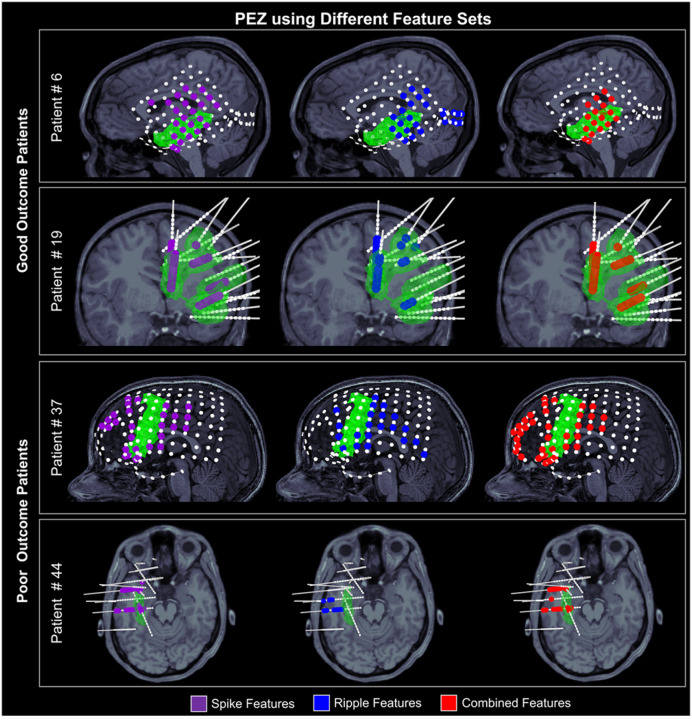
Case studies of good and poor outcome patients. **a** Predicted epileptogenic zone (PEZ) from spike (SF, purple), ripple (RF, blue), and combined (CF, red) feature-based Random Forest classifiers in two good outcome patients (#6 and #19) and two poor outcome patients (#37 and #45). Green areas in the figures represent resection and white circles represent contacts.

To illustrate how the PEZ relates to the EZ, we report findings from four representative cases: two patients with good and two patients with poor surgical outcome. For these patients, we depict the PEZ overlayed on their MRIs and the resection volume (Fig. 5). For good outcome patient #6, the PEZ derived from the CF (*O*_*RES*_= 71%) and SF (*O*_*RES*_= 51%) showed greater overlap with the resection volume than that from the RF (*O*_*RES*_= 41%) (Fig. 5). The PEZs from the CF and SF were also more focal (*F*_*PEZ*_=22 mm and 23 mm, respectively) and closer to the resection (*D*_*RES*_=12 mm and 13 mm, respectively) compared to the RF (*F*_*PEZ*_=32 mm, *D*_*RES*_=19 mm). For good outcome patient #19, PEZs from all three classifiers (i.e., SF, RF, and CF) had high overlap with the resection (*O*_*RES*_>85%), were focal (*F*_*PEZ*_ <23 mm), and close to the resection (<4mm) (Fig. 5).

In contrast, for the poor outcome patient #37, the PEZs from all three classifiers showed low overlap with resection (*O*_*RES*_ < *34%*) and low focality (*F*_*PEZ*_ >31mm). Finally, for poor outcome patient #44, although the PEZs were focal (*F*_*PEZ*_<20mm), they showed a low overlap with the resection (*O*_*RES*_ < *41%*) (Fig. 5).

## Discussion

In this study, we introduce a novel supervised ML-based framework that delineates the EZ and predicts the surgical outcome in patients with DRE by extracting and combining features of interictal biomarkers from iEEG data in a fully automated fashion. Our framework detects spikes and ripples, extracts relevant temporal, spectral, and spatial features, and employs Random Forest classifiers to identify the PEZ. We evaluated the performance of both the individual features and Random Forest classifiers trained on SF, RF, and CF with RES as target. Our findings indicate that the SF and CF classifiers not only outperformed the models based on individual feature sets but also yielded a focal PEZ with high overlap and proximity to the resection in patients with good surgical outcome. Moreover, resection of the PEZ, defined by the CF, achieved an accuracy of ~ 80% in predicting surgical outcome, while resection of the clinically defined SOZ showed limited predictive value. This discrepancy likely reflects fundamental limitations of the SOZ concept in sEEG and depth-electrode-based evaluations, where seizure onset may be rapidly distributed across mesial and lateral temporal structures^66^, or reflect only the early propagation rather than the full epileptogenic network.^67,68^ In addition, the SOZ is constrained by electrode coverage of the EZ, which may incompletely capture deeper or spatially remote regions contributing to seizure generation. As a result, SOZ localization alone may fail to identify all tissue necessary for resection and achievement of seizure freedom. Our framework generalizes across different implantation types and enables fully automated epileptogenic zone localization from interictal iEEG data, substantially reducing the need for manual identification and evaluation of epileptogenic brain regions.

### Spike spatial distribution plays a key role in defining the EZ

Interictal biomarkers such as spikes and ripples, as well as their features including event rate, power, spike-ripple co-occurrence and their onsets, have been previously associated with the EZ.^11,58,69–72^ Our results were consistent with these previous findings where several spike and ripple features had higher values within the resection (compared to outside) for good-outcome patients (Fig. 2a). Notably, some features also showed elevated values within resection for patients with poor outcome but with lower effect size (Fig. 2b). These findings indicate that although each feature independently captures distinct aspects of the EZ, resection of brain regions based on individual features alone does not necessarily lead to seizure freedom. Upon ranking feature importance, distance-based spike features emerged as the most informative (showing higher effect sizes) than other features in distinguishing epileptogenic from non-epileptogenic contacts (Fig. 2b). This notion was further validated by the EZ prediction analysis, where performance metrics showed that the ED-SO, ED-HSR, and ED-HSP achieved higher AUC-ROC, AUC-PR, and *O*_*RES*_ values compared to the other features (Fig. 2b). This aligns with a recent study showing that the spatial distribution of biomarkers plays a key role in defining the EZ.^48^

### Combined Features define the EZ better than individual features

While each feature captures a distinct characteristic of the epileptogenic tissue, no single measure fully represents the complex spatial, spectral, and temporal dynamics of the EZ. A substantial body of literature suggests that ML-based integration of multiple electrophysiological biomarkers yields a more accurate localization of the EZ compared to individual features alone.^52,53,56,57,73,74^ Such integration facilitates the discrimination between epileptogenic and non-epileptogenic contacts, even when measurements are incomplete or noisy. Our findings support this notion, as SF and CF classifiers outperformed individual features when predicting the epileptogenic and non-epileptogenic contacts. Specifically, SF and CF outperformed all individual features in terms of AUC-ROC and AUC-PR (except for the ED-HSR, ED-HSP, and ED-SO) (Fig. 3a). Moreover, SF and CF exhibited higher *O*_*RES*_ than all individual features, except for the SRC (Fig. 3b). Overall, our results highlight the added value of integrating multiple spatial, temporal, and spectral interictal features to improve the delineation of PEZ and support the notion that ML classifiers can capture nonlinear feature relationships of different epileptogenic activity more effectively than single-feature approaches.

### Ripple features provide modest improvement over spike features

The Random Forest classifiers trained using the CF slightly outperformed those trained on SF, suggesting that spike features capture most of the relevant epileptogenic activity (Fig. 3). While combining spike and ripple features provided a more accurate representation of the PEZ than any individual feature alone, our results indicate that ripple features provided only a modest improvement over spike-based features. This may be due to the fact that not all ripples are pathological, underscoring the importance of distinguishing pathological from physiological ripples, which also occur in non-epileptogenic brain regions and during normal brain fucntion.^75,76^ Although efforts have been made to address this distinction suggesting that pathological HFOs tend to co-occur with spikes and exhibit shorter durations and lower power and amplitude^22,75,77,78^, other studies reported the opposing findings.^79,80^ Collectively, our findings suggest that, although integrating multiple biomarkers can offer incremental gains, spike features remain the dominant contributor to accurate identification of the PEZ based on interictal data. These findings are consistent with a recent study showing that low-frequency features alone were sufficient for the accurate delineation of the EZ and prediction of surgical outcome, with high-frequency features providing little or no additional value.^53^

### Combined Features predict outcome with higher accuracy than individual features

Previous studies have reported strong associations between interictal biomarkers and postsurgical outcome.^11,22,37,81–86^ These results indicate that, in patients with focal epilepsy, seizure-generating regions form a distinct pathological network isolated from surrounding brain tissue. When these pathological regions are completely resected, patients are more likely to achieve postoperative seizure freedom. Indeed, the spatial concordance between the identified epileptogenic regions and the resected zone has previously been used as a reliable predictor of success.^37,40,82,85,87^ Our findings support this notion since we observed a decrease in the *O*_*RES*_ as well as increased *D*_*RES*_ in poor outcome patients (Fig. 4). These findings may indicate the presence of other epileptogenic regions distant from the resection, which may not have been resected due to overlap of the EZ with eloquent areas or could have been entirely missed [e.g., patient #37 and #44 (Fig. 5)]. Upon assessing the ability of individual features to predict surgical outcome, we found that almost all features were predictive, except for the SPI, RR, and RPI (Fig. 2b). All Random Forest classifiers were predictive of the surgical outcome (Fig. 4b). When comparing outcome predictive performance across all individual features and classifiers, CF outperformed many of the features but not SR, ED-HSR, ED-HSP, ED-SO, ED-HRP, SF, and RF. These findings suggest that outcome prediction remains feasible using individual features alone, and that combining features does not necessarily improve performance, as many individual features are already informative (Fig. 4c).

### Limitations

Despite these promising results, several limitations should be acknowledged. Our retrospective study analyzed iEEG data from a single-center cohort of patients with DRE and heterogeneous pathologies. Because the models were trained using data from good outcome patients, future studies from larger, multicenter cohorts are needed to evaluate its clinical applicability, particularly within more homogeneous patient populations.

Resection volumes from good outcome patients are often used as a gold standard for the EZ, despite the possibility that they include non-epileptogenic tissue.^1,88^ This may result in either missing critical epileptogenic regions or defining areas larger than the actual EZ. Because ML model performance depends strongly on the accuracy of the ground truth used, improved approximations of the EZ are needed to provide more reliable targets for ML training. Moreover, iEEG is spatially limited since it is based on a subjective hypothesis agreed upon during presurgical evaluation. Consequently, the EZ was likely sampled well in good outcome patients but may have been missed in poor outcome patients. Advances in whole-brain iEEG implantations may overcome these drawbacks. Furthermore, future studies on longer data segments may be conducted to enhance the accuracy of PEZ in delineating the EZ. Finally, the retrospective nature of the study warrants further validation in larger, prospective cohorts to confirm the robustness and generalizability of these findings.

## Conclusion

In this study, we systematically evaluated the predictive power of individual features and combined feature-based classifiers for localizing the EZ and predicting surgical outcome in patients with DRE. Our results demonstrate that Random Forest classifiers outperform individual features, providing more focal and accurate identification of the EZ. While spike features remain highly informative for outcome prediction, the integration of ripple features offers a modest additional advantage. Importantly, these findings support the use of short segments of interictal data to approximate the EZ and inform resection planning, potentially reducing reliance on ictal recordings. Future prospective studies with larger cohorts are necessary to validate and refine this approach for clinical translation.

## Materials and Methods

### Patient Cohort

We retrospectively analyzed iEEG data of patients with DRE, from Boston Children’s Hospital (BCH, 51 patients, admitted between June 2011 and June 2023) and Cook Children’s Medical Center (CCMC, 11 patients, admitted between December 2019 and August 2021). The patients had long-term monitoring with iEEG and underwent resection neurosurgery. The selection of the patients was based on the following criteria: (i) availability of at least 5-minute interictal iEEG epochs with no biological or technical artifacts; (ii) availability of post-implantation computerized tomography (CT); (iii) availability of preoperative and post-operative MRIs; (iv) accurate information about the resection volume, resected contacts, and the clinically defined SOZ; (v) availability of post-surgical outcome at least one year after surgery; (vi) data containing spikes and ripples; (vii) sampling frequency > 1,000 Hz. The surgical outcome was determined by a pediatric epileptologist after multiple follow-up visits. We used the Engel score^89^ to classify patients as good (Engel 1) or poor outcome (Engel 2–4). The patient demographic and the clinical information of the cohort are provided in Table 1. The protocol was approved by North Texas Regional IRB (2019 − 166; PI: C. Papadelis), that waived the need for informed consent considering the study’s retrospective nature. All methods and analyses were performed in accordance with relevant guidelines and regulations.

### Interictal iEEG recordings

At BCH, long-term iEEG data were collected using stereotactic electrodes, subdural grids, or combined subdural grids and depth electrodes utilizing XLTEK Quantum NeuroWorks (Natus Inc., USA). The subdural electrodes had a diameter of 2–3 mm with a 10 mm inter-contact spacing, while the depth electrodes consisted of 6 to 16 contacts with a diameter of 0.8 mm and were linearly arranged approximately 3–5 mm apart. At CCMC, long-term iEEG data were collected using stereotactic electrodes utilizing Quantum NeuroWorks (Natus Inc., USA). The electrodes consisted of 8 to 16 contacts with a diameter of 0.8 mm and were linearly arranged approximately 3–5 mm apart.

The data were recorded with a sampling frequency of 1,000 to 2,048 Hz. We specifically chose 5-minute interictal segments during non-REM slow-wave sleep (when applicable), occurring at least one hour before or after clinical seizures or half an hour before or after an electrographic seizure.^90^ This approach ensured the incorporation of epochs with the highest spike rate and minimal motion artifacts.^91,92^ Noisy channels and short segments with artifacts were excluded.

### Structural Imaging and iEEG recordings

MRI scans utilizing standard magnetization-prepared rapid acquisition gradient-echo sequences were performed both before and following resection with a 3T scanner (TIM TRIO, Siemens AG at BCH and Magnetom Skyra at CCMC). Post-implantation CT scans, with voxel size set at 0.5 × 0.5 × 0.5 mm^3^, were performed after iEEG electrode implantation to document electrode locations. We aligned the electrode coordinates by registering the post-implantation CT scans with the preoperative MRI using *Brainstorm*.^93^ We then projected the electrode locations onto the patient’s cortical surface reconstructed from their preoperative MRI with *Freesurfer*.^94^ We further adjusted the stereotactic and depth electrode positions to accommodate for brain shifting and pneumocephalus post-electrocorticography implantation.^95^ The number and type of implanted electrodes for each patient are reported in **Supplementary Tables 1 and 2**.

### Defining the SOZ and resection contacts

For each patient, pediatric epileptologists determined the SOZ by visually examining ictal data. iEEG contacts that exhibited altered dynamics at the onset of seizures were designated as SOZ contacts. This process was conducted independently of the current study. To delineate the resection volume, we registered the preoperative and postoperative MRIs and manually outlined the boundaries of the resection volume on sequential slices using *Brainstorm*^93^ and MATLAB’s *volumeSegmentor* toolbox. Subsequently, contacts ≤ 10 mm from resection were considered as resected (RES).

### Spike and Ripple Features

To generate the features used in our classifiers, we focused on quantifying the spectral and spatiotemporal characteristics of spike and ripple propagations. We developed an in-house MATLAB algorithm to identify propagation sequences of spikes and ripples separately across all iEEG contacts for each patient.^65^ After detecting the spikes, we employed our algorithm that marks the earliest spike within each sequence as onset (Fig. 1b).^11^ Then, it adds the next spike to the propagation sequence if it occurred within a predefined time window, i.e. the inter-event latency. The contacts in every propagation were indexed sequentially, starting from one, denoting the onset contact, followed by increasing numbers for the subsequent spikes. A similar approach was adopted for mapping ripple propagations (Fig. 1c). Previous studies have shown that the inter-event latency for spikes and ripples is in the range of 3–15 ms^10,96^ and 10–32 ms^10,42^, respectively. Based on these findings, we set here the inter-event latency to 10 and 30 ms, to map spike and ripple propagations respectively.^11^ We also discarded propagations if more than 50% of their events occurred within 2 ms from each other.^52,97^ Using spikes and their propagation events, we computed the following features for each channel (Fig. 1d–f): (i) spike rate (SR); (ii) average spike power (SP); (iii) median spike propagation index (SPI); (iv) average Euclidean distance of contacts from the contact with the highest spike rate (ED-HSR); (v) average Euclidean distance of contacts from the contact with the highest average spike power (ED-HSP); and (vi) average Euclidean distance of the contacts from spike onset contact (ED-SO). Similarly, using ripples and their propagation events, we computed for each channel the: (i) ripple rate (RR); (ii) average ripple power (RP); (iii) median ripple propagation index (RPI); (iv) average Euclidean distance of contacts from the contact with the highest ripple rate (ED-HRR); (v) average Euclidean distance of contacts from the contact with the highest ripple power (ED-HRP); and (vi) average Euclidean distance of the contacts from ripple onset contact (ED-RO). Finally, we identified the cooccurrence of spikes and ripples in each contact and computed the average spike-ripple cooccurrence rate (SRC) per channel. Table 2 summarizes the features adopted in this study.

We normalized the SR, RR, SP, RP and SRC between 0 and 1 using Min-Max scaling ensuring that the minimum value is scaled to 0 and the maximum to 1. For a feature *x*, the min-max normalized value *x′* is defined by:

1 $$x\prime =\frac{x-x_{min}}{x_{max}-x_{min}}$$

where *x*_*min*_ is the minimum value and *x*_*max*_ is the maximum.

The SPI, RPI, ED-HSR, ED-HRR, ED-HSP, ED-HRP, ED-SO, and ED-RO were scaled using Max-Min scaling ensuring that the maximum value is scaled to 0 and the minimum to 1. For a feature *x*, the max-min normalized value *x′* is defined by:

2 $$x\prime =\frac{x_{max}-x}{x_{max}-x_{min}}$$

### Performance of individual features in EZ and outcome prediction

For each feature, we estimated and compared the normalized values inside and outside the SOZ and resection for good and poor outcome patients, separately (*Wilcoxon signed-rank* test).

Then, to further evaluate the predictive power of individual features, we used the normalized values of each feature as predictors and RES as target. We performed the analysis only for good outcome patients since their resected tissue is assumed to contain the actual EZ. First, we assessed feature importance using one-way ANOVA. For each feature, we quantified the proportion of variance explained by the class labels (η^2^)^98^, which provides a measure of how strongly a feature differentiates between classes (i.e. epileptogenic vs. non-epileptogenic). Values of η^2^ greater than 0.06 generally indicate that a feature has a meaningful influence on distinguishing between epileptogenic and non-epileptogenic classes. Next, for each feature, we used the LOPO-CV approach to assess its ability to predict the RES. At each step, one patient was left out, and the remaining patients’ feature values were collectively used to compute the optimal thresholds based on the maximum Youden index (J) obtained from the ROC curves. Then, the left-out patient’s features were thresholded using the computed optimal threshold to identify PEZ. We then defined the following: (i) true positives (TP), PEZ contacts that were located within the RES; (ii) false positives (FP), PEZ contacts that were located outside the RES; (iii) false negatives (FN), non-PEZ contacts that were located inside the RES; and (iv) true negatives (TN), non-PEZ contacts that were located outside the RES. We then calculated the following performance metrics: (i) sensitivity [TP/(TP + FN)]; (ii) specificity [TN/(TN + FP)]; (iii) precision [TP/(TP + FP)]; (iv) NPV [TN/(TN + FN)]; (v) accuracy [(TP + TN)/(TP + TN + FP + FN)]; (vi) F1-score [2 × TP/(2 × TP + FP + FN)]; (vii) AUC-ROC; (viii) AUC-PR. The overlap with resection (*O*_*RES*_) was defined as the percentage of PEZ contacts within a 10 mm distance from the resection. The selection of the 10 mm cut-off was based on studies that showed that the gyral width is between 11–21 mm.^99^ We hypothesized that the resection of PEZ is linked to favorable surgical outcome, thus, we considered *O*_*RES*_ as predictor and the dichotomized patient’s outcome as target. We considered: (i) TP, the number of good outcome patients who had ≥ 50% of the PEZ resected (*O*_*RES*_ ≥ 50%); (ii) FN, the number of good outcome patients who had < 50% of the PEZ resected (*O*_*RES*_ < 50%); (iii) FP, the number of poor outcome patients who had ≥ 50% of the PEZ resected; (iv) TN, the number of poor outcome patients who had < 50% of the PEZ resected. We then constructed the confusion matrices for each feature and computed the *Fisher’s exact* test *p*-values to find significance.

### Training and evaluation of Random Forest classifiers

We trained and compared the performance of different Random Forest classifiers using different subsets of predictors and targets. We considered three sets of features as predictors: spike-features only (SF), ripple-features only (RF), and combined features (CF). We used RES to approximate the EZ. The setup resulted in training and evaluating three different classifiers with different combination of predictors (i.e., SF, RF, and CF). We exclusively trained and evaluated our classifiers using only patients with good outcome, since these patients remained seizure-free post-surgery, indicating successful removal of the EZ. Regarding the Random Forest classifier’s number of trees parameter, we conducted a five-fold cross-validation, varying the number of trees from 10 to 500 and selecting the value that yielded a stable AUC-ROC performance. Based on this analysis, we set the number of trees to 100 (**Supplementary Note 2**).

Classifier performance was evaluated using TP, TN, FP, and FN definitions identical to those used for individual feature analyses. Subsequently, we trained and tested the performance of the Random Forest classifier using LOPO-CV and computed performance metrics (i.e., sensitivity, specificity, precision, NPV, accuracy, F1-score, AUC-ROC, and AUC-PR). After estimating the PEZ using the trained classifiers, we computed *F*_*PEZ*_, *O*_*RES*_, and *D*_*RES*_. While training was performed using only good outcome patients, testing included both good and poor outcome patients.

### Robustness of PEZ across implantation types

To evaluate whether our framework provides consistent findings among different implantation types, we examined the ability of PEZ to predict the EZ in good outcome patients with different implantation types. We first divided our good outcome patients into three subsets; (i) ECoG, patients with subdural implantations; (ii) sEEG, patients with stereotactic implantations; and (iii) ECoG and depth, patients with ECoG and a few depth electrodes (**Supplementary Table 1**). For each subset, we trained and tested using CF with LOPO-CV and computed performance metrics (sensitivity, specificity, precision, NPV, accuracy, F1-score, AUC-ROC, AUC-PR) along with spatial properties (*O*_*RES*_, Dice score, *D*_*RES*_, *F*_*PEZ*_) of the PEZ.

### Random forest classifiers performance to predict surgical outcome

We presumed that the *O*_*RES*_ of PEZ predicted from the classifiers SF, RF, and CF could predict outcome. Specifically, we assumed that in patients with good outcome, the PEZ would be located within resection, whereas in those with poor outcome, the resection would have missed the EZ. Subsequently, we performed a prediction analysis where we used the *O*_*RES*_ of the PEZ as predictor of outcome. We considered good and poor outcomes as positive and negative classes, respectively. We then defined: (i) TP, the number of good outcome patients who had ≥ 50% of the PEZ resected (*O*_*RES*_ ≥ 50%); (ii) FN, the number of good outcome patients who had < 50% of the PEZ resected (*O*_*RES*_ < 50%); (iii) FP, the number of poor outcome patients who had ≥ 50% of the PEZ resected; and (iv) TN, the number of poor outcome patients who had < 50% of the PEZ resected. We constructed confusion matrices for each feature set and computed the Fisher’s exact test *P*-values to assess significance. Finally, we computed the sensitivity, specificity, precision, NPV, F1-score, and accuracy of the outcome predictions.

### Statistical Analysis

The Kolmogorov-Smirnov test was used to test the normality of features. *Cliff’s d* measure was used to compute effect sizes. Demographic data and PEZ spatial measures (*F*_*PEZ*_, *O*_*RES*_, Dice score, and *D*_*RES*_) are reported as median (25th −75th percentile). The *chi-squared* test was used to compare the effect of different cohort characteristics (i.e., gender, implantation side, epilepsy localization, and MRI findings) on outcome. We applied two-sided non-parametric *Wilcoxon signed-rank* test for all paired comparisons (median feature values inside vs. outside the SOZ and resection). We applied the two-sided *Wilcoxon rank-sum* test for non-paired comparisons between good and poor outcome patients. Bonferroni correction was applied in all multiple comparison tests.

We used the one-sided *Fisher’s exact* test to evaluate the predictive value of the PEZs to predict outcome. To evaluate the performance of all individual features and trained Random Forest classifier models, we performed a comparative analysis of EZ prediction using the AUC-ROC, AUC-PR, and *O*_*RES*_. Pairwise comparisons were performed using the *Wilcoxon signed-rank* test on each performance metric, trained and evaluated using the LOPO-CV approach. False discovery Rate (FDR) was applied to correct for multiple comparisons. The results are displayed in a square matrix where each element indicates whether a significant difference (*P* < 0.05) between the different predictor models was observed and, if so, identifies the model with the higher median value.

For outcome prediction using individual features, classifier models, and SOZ, we performed a pairwise comparison using the *McNemar* test. The elements in the corresponding square matrix show both the presence of significant differences (*P* < 0.05) and the ID of the model with higher accuracy. The McNemar test was used to examine differences in the ability to predict outcomes using confusion matrices between different features and the proposed ML classifiers. All analysis was performed with MATLAB 2023b (The MathWorks, Inc.).

## Supplementary Material

This is a list of supplementary files associated with this preprint. Click to download.

- SupplementaryMaterial.docx

## Figures and Tables

**Table 1:** Patient demographic information categorized by post-surgical outcome.

Characteristic	Total	Good outcome (Engel = I)	Poor outcome (Engel > I)	*p*
**Patients**	62	34	28	-
**Gender**				0.17^a^
Male	31	18	13	
Female	31	16	15	
**Age at surgery**, yr., median (IQR).	12 (8–15.5)	12 (7–15.5)	12 (8–15)	0.8^b^
**Age at seizure onset**, yr., median (IQR).	4.5 (2–9)	6 (2–10.25)	4 (2–6.5)	0.08^b^
**Follow up**, yr., median (IQR).	2.5 (2–5)	2.5 (2–5)	2.5 (1–5)	0.8^b^
**Number of contacts**, no., median (IQR)	124 (102–151)	112 (88–136)	138 (106–168)	0.09^b^
**Type of implantations**				0.73^a^
Subdural (ECoG)	13	8	5	
sEEG	36	20	16	
Subdural and depth	13	6	7	
**Implantation side**				0.96^a^
L	40	22	18	
R	16	9	7	
L+R	6	3	3	
**Eplilepsy localization**				0.16^a^
Temporal	26	17	9	
Extra-temporal	36	17	19	
**MRI Findings**				0.73^a^
NL	17	9	8
MCD	34	20	14
ACQ	11	5	6

ACQ = Acquired (i.e., stroke, neoplasm, and traumatic brain injury); ECoG = Electrocorticography; IQR = Inter-Quartile Range; L = Left; MCD = Malformation of Cortical Development (i.e., focal cortical dysplasia, polymicrogyria, gliosis, and tuberous sclerosis complex); NL = Non-Lesional; no. = Number; *p* = *p*-values; R = Right; sEEG = stereotactic electroencephalography; yr. = Year.

a *Chi-squared* test;

b *Wilcoxon rank-sum* test (two-tailed).

**Table 2:** Summary of spike and ripple features used for EZ characterization.

Biomarker	Feature	Description	Type	Normalization	Physiological Meaning
**Spike** (1–70 Hz)	**SR**	Spike rate (min^−1^)	Temporal	Min–Max	Interictal spike excitability
	**SP**	Spike power	Spectral	Min–Max	Signal energy
	**SPI**	Spike propagation index	Temporal	Max-Min	Degree of spike spread across channels
	**ED-HSR**	Euclidean distance from highest spike rate contact	Spatial	Max-Min	Proximity to maximal spike-generating region
	**ED-HSP**	Euclidean distance from highest spike power contact	Spatial	Max-Min	Proximity to strongest spike source
	**ED-SO**	Euclidean distance from spike onset contact	Spatial	Max-Min	Distance from earliest spike onset
**Ripple** (80–250 Hz)	**RR**	Ripple rate (min^−1^)	Temporal	Min–Max	High-frequency excitability
	**RP**	Ripple power	Spectral	Min–Max	Signal energy
	**RPI**	Ripple propagation index	Temporal	Max-Min	Spatial spread of ripple activity
	**ED-HRR**	Euclidean distance from highest ripple rate contact	Spatial	Max-Min	Proximity to dominant ripple-generating region
	**ED-HRP**	Euclidean distance from highest ripple power contact	Spatial	Max-Min	Proximity to strongest ripple source
	**ED-RO**	Euclidean distance from ripple onset contact	Spatial	Max-Min	Distance from earliest ripple onset
**Spike–Ripple**	**SRC**	Spike–ripple co-occurrence rate	Temporal	Min–Max	Pathological coupling of spikes and ripples

## Data Availability

The data are available from the corresponding author upon request.

## Code availability

The code is available from the corresponding author upon request.
